# Curvature Matters: Modeling Calcium Binding to Neutral and Anionic Phospholipid Bilayers

**DOI:** 10.1021/acs.jpcb.3c01962

**Published:** 2023-05-16

**Authors:** Semen Yesylevskyy, Hector Martinez-Seara, Pavel Jungwirth

**Affiliations:** † Institute of Organic Chemistry and Biochemistry, Academy of Sciences of the Czech Republic, Flemingovo náměstí 542/2, 160 00 Prague 6, Czech Republic; ‡ Department of Physics of Biological Systems, Institute of Physics of the National Academy of Sciences of Ukraine, Nauky Avenue 46, 03038 Kyiv, Ukraine; § Receptor.AI Incorporated, 20-22 Wenlock Road, N1 7GU London, U.K.

## Abstract

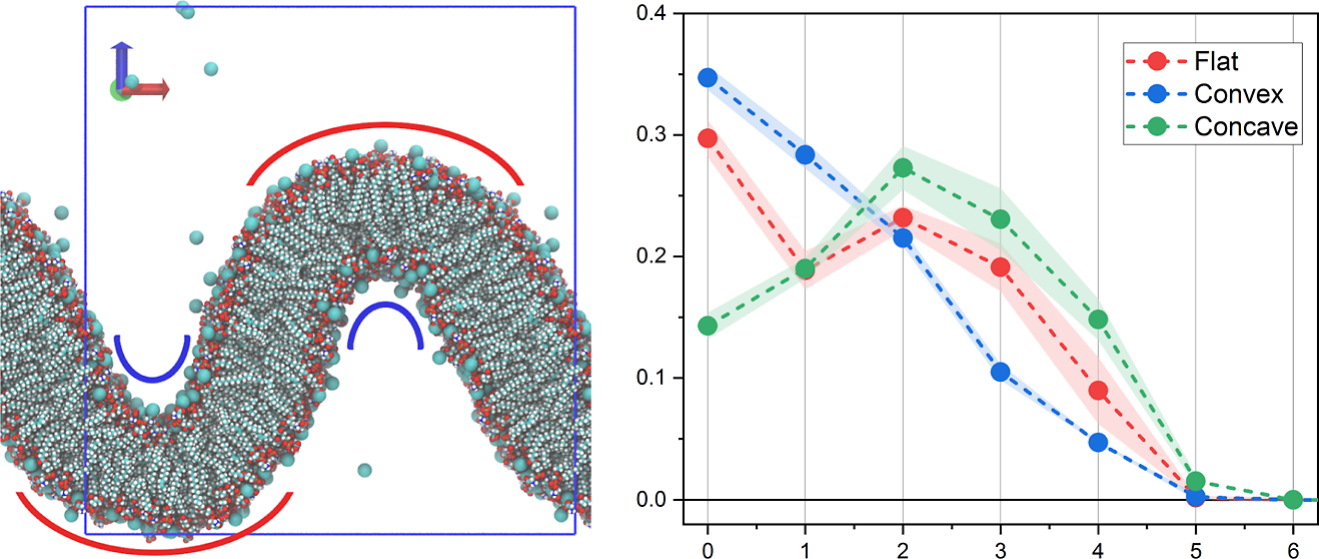

In this work, the influence of membrane curvature on the Ca^2+^ binding to phospholipid bilayers is investigated by means of molecular dynamics simulations. In particular, we compared Ca^2+^ binding to flat, elastically buckled, or uniformly bent zwitterionic and anionic phospholipid bilayers. We demonstrate that Ca^2+^ ions bind preferably to the concave membrane surfaces in both types of bilayers. We also show that the membrane curvature leads to pronounced changes in Ca^2+^ binding including differences in free ion concentrations, lipid coordination distributions, and the patterns of ion binding to different chemical groups of lipids. Moreover, these effects differ substantially for the concave and convex membrane monolayers. Comparison between force fields with either full or scaled charges indicates that charge scaling results in reduction of the Ca^2+^ binding to curved phosphatidylserine bilayers, while for phosphatidylcholine membranes, calcium binds only weakly for both force fields.

## 1 Introduction

Calcium plays a crucial role in a number of biological processes, including formation of bones, teeth, and connective tissues, as well as in blood clotting or enzymatic catalysis. Calcium ions also regulate a plethora of intracellular signaling and metabolic pathways, which are involved in a broad range of cellular functions ranging from neurotransmission and muscle contraction to apoptosis and cell division.^1^

Calcium ions are known to bind strongly to lipid bilayers containing anionic lipids. This results in the increase of general membrane rigidity and ordering,^2,3^ conformational changes of the lipid head groups,^4^ ordering of the lipid tails,^4^ and the decrease of lipid hydration.^5^ The binding of Ca^2+^ ions to the cellular membranes is known to be crucial for calcium signaling pathways,^6,7^ the functioning of membrane proteins,^8,9^ membrane remodeling and fusion processes,^10−12^ and modulation of the membrane permeability.^13^

Although multiple experimental techniques are available to assess Ca^2+^ interaction with lipid membranes,^14−17^ they are inevitably limited by spatial and temporal resolution. These techniques often lack complete control over lipid composition, membrane shape and topology, and environmental conditions. In this situation, molecular dynamics (MD) simulations may prove invaluable for studying Ca^2+^ interactions with the membranes.^18^

Calcium ions are parameterized in most existing force fields, which are routinely used for simulating various Ca^2+^-containing systems. However, the widely used classical non-polarizable force fields tend to overestimate the interactions of Ca^2+^ with proteins and peptides,^1,19−24^ phospholipids,^10,25−28^ and nucleic acids^29,30^ to the extent that raises a question of their practical applicability for quantitative assessment of calcium binding to any biologically relevant molecule.

Several techniques were proposed to overcome this overbinding problem, such as the NBFIX correction^28,31^ that aims at reducing the electrostatic attraction by effectively increasing the ionic size. Another approach is introduction of the multi-site models, which add charged dummy particles to the Ca^2+^ ion.^32,33^ Although these models reproduce the first solvation shell of Ca^2+^ ions correctly and performs well for calcium binding to proteins and RNA, they were not tested for lipid bilayers and introduce artificial anisotropy to Ca^2+^ coordination.

Recent development of the scaled-charge force fields within the electronic continuum correction (ECC) approach provides an elegant way of addressing the calcium overbinding problem without sacrificing the computational efficiency and without additional parameters for the Ca^2+^ model.^1^ This approach implicitly accounts for electronic polarization effects in a mean-field way by scaling the partial charges of interacting atoms in charged molecular groups. It was shown that the force fields based on ECC, particularly prosECCo75,^1,34^ are superior to non-polarizable force fields in the description of Ca^2+^ binding to peptides and lipid bilayers.^27^

One of the intrinsic properties of the lipid bilayers in living cells often overlooked in simulations is their curvature. Indeed, cellular plasma membranes and the membranes of different organelles exhibit a wide variety of shapes ranging from flat surfaces to highly curved protrusions, invaginations, vesicles, and tubules. The causes of membrane curvature are diverse,^35^ ranging from spontaneous curvatures of the lipids^36^ to the effect of scaffolding protein domains^37^ or a mechanical influence of the cytoskeleton.^38^ The membrane curvature has a well-recognized role in many cellular processes, such as vesicle budding, membrane fusion, lipid and protein sorting, and enzyme activation.^38^

There has been a growing interest in molecular simulations of non-planar membranes in recent years, resulting in the rapid development of novel techniques for creating,^39^ maintaining,^40^ and analyzing^41−43^ the membrane curvature. Despite the emergence of such tools, detailed studies of the ions binding to curved lipid bilayers are still lacking.

Curved lipid bilayers containing anionic lipids are particularly interesting in this respect. Indeed, calcium ions are known to coordinate and cross-link anionic lipid head groups, while the changes in membrane curvature modulate the environment for such cross-linking. The lipid head groups are congested in concave membrane leaflets, and the ions are confined between them. In contrast, in convex leaflets, the lipids are further apart, and the coordination of ions by lipids decreases. This difference creates a heterogeneous and diverse environment for Ca^2+^ binding in the same chemical systems which has not been explored in detail yet.

In this work, we systematically investigate the binding of Ca^2+^ to curved phosphatidylcholine (POPC) and phosphatidylserine (POPS) lipid bilayers. First, we investigate Ca^2+^ binding to elastically buckled POPC and POPS membranes with heterogeneous and fluctuating curvature. Next, we perform a detailed analysis of Ca^2+^ binding and coordination in a POPS membrane with a uniform curvature. Finally, we compare results employing force fields with full or scaled charges.

## 2 Methods

### 2.1 Membrane Buckling

#### 2.1.1 System Preparation

The POPC and POPS membranes were created using the CHARMM-GUI Membrane Builder^44,45^ as periodic patches with 128 lipids (64 per monolayer) solvated with 50 water molecules per lipid.

The CHARMM36 force field with the NBFIX correction for selected Ca^2+^ interactions used has been employed here.^28^ Gromacs parameter files generated by the CHARMM-GUI Membrane Builder module include NBFIX automatically if any of the relevant atom types is found in the system. In our case, the NBFIX non-bond parameters are explicitly generated for the following pairs of atom types: CAL-CLA, CAL-O2L, and CAL-OCL.

For initial buckling simulations, the CHARMM36 force field with the NBFIX correction for Ca^2+^ was used.^28,31^ Since no Ca^2+^ ions were present in the system at this stage, there is no detectable difference between CHARMM36 and prosECCo75 force fields. The K^+^ neutralizing counter ions were added to the POPS system. The membrane patches were equilibrated for 100 ns with the standard semi-isotropic pressure coupling. Equilibrated bilayer patches were used as the planar reference systems in production simulations.

The systems were then multiplied in the *X*-direction to get elongated membrane patches with 640 lipids (320 in each monolayer). The slab of water was added in the *Z*-direction to reach the final solvation of ∼104 water molecules per lipid. The obtained systems were subject to equilibration with anisotropic pressure coupling along the *X* and *Z*-directions with reference pressures of 1000 and 1 bar, respectively. This leads to gradual squeezing of the simulation box over *X* with its simultaneous expansion over *Z* and to the elastic buckling of the membrane. The simulations were stopped upon reaching the *X*-box size of ∼20.5 nm. After that, the box size was fixed in the *XZ* plane, and the pressure coupling along the *Y* was applied with the reference pressure of 1 bar.

#### 2.1.2 Production Simulations

**Figure 1:**
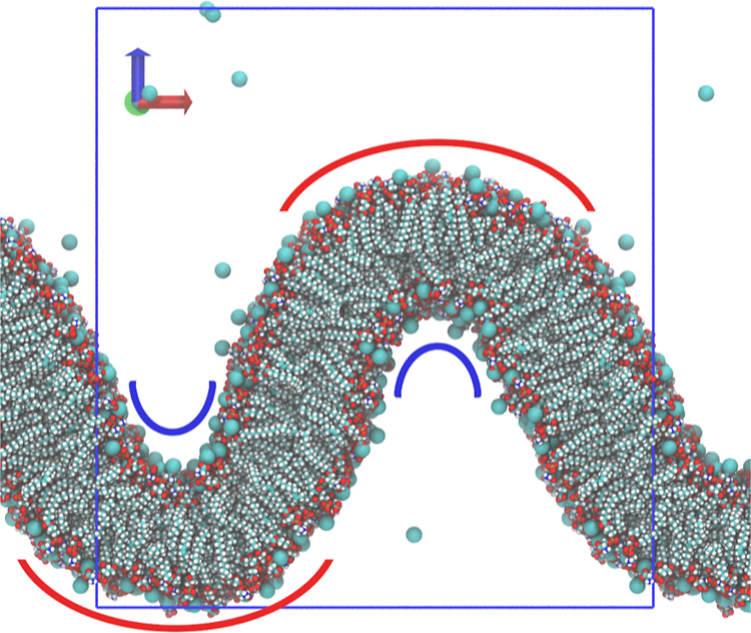
Snapshot of the equilibrated buckled POPS bilayer. Ca^2+^ ions are shown as spheres, and water is not shown for clarity. Blue rectangle represents the simulation box. Blue arches denote the concave membrane regions with highly negative curvature, while red arches show convex membrane regions with a positive curvature.

At this stage, 320 Ca^2+^ ions (1:2 ion-to-lipid ratio) were added to the POPC system and neutralized with Cl^–^ ions. For the POPS system, 320 K^+^ counter ions, which were used for equilibration, were randomly substituted by Ca^2+^, while the rest of them were deleted. Each system was simulated independently with the prosECCo75 force field^1^ for 300 ns. The last 200 ns was used for analysis. Additionally, the same simulations using CHARMM36 with the NBFIX correction for Ca^2+^^28,31^ were performed for comparison. Unless indicated otherwise, the results presented in the main text always correspond to the prosECCo75 force field. A snapshot of an equilibrated buckled POPS membrane is shown in Figure 1. Finally, planar reference bilayers were simulated, preserving the same 1:2 ion-to-lipid ratio.

#### 2.1.3 Technical Details

All MD simulations were performed using the program Gromacs^46^ version 2021.4 at the pressure of 1 atm maintained by the Parrinello–Rahman barostat.^47^ The velocity rescale thermostat^48^ was used with a reference temperature of 300 K. The Verlet cutoff scheme was used.^49^ Force-switch cutoff of the van der Waals interactions was used between 1.0 and 1.2 nm. Long-range electrostatics was computed with the particle mesh Ewald method^50^ with the cutoff of explicit short-range electrostatic interactions at 1.2 nm. An integration step of 2 fs was used in all simulations, with the bonds to hydrogen atoms converted to rigid constraints using the LINCS algorithm.^51^

#### 2.1.4 Analysis of the Curvature

**Figure 2:**
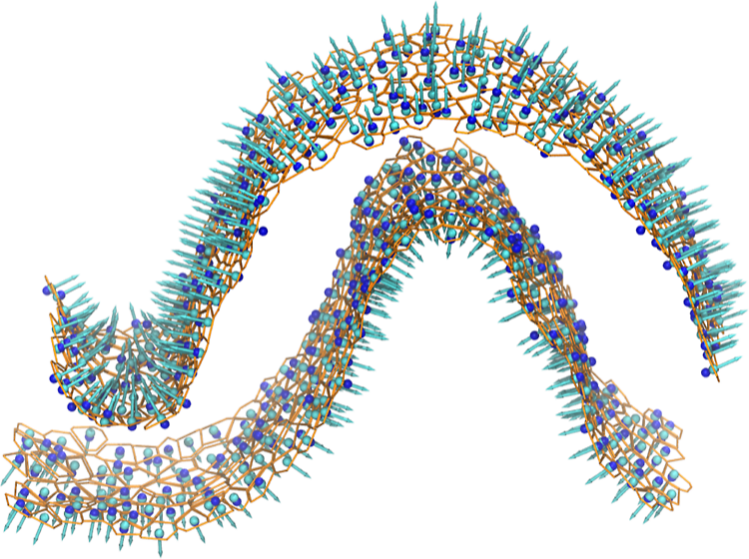
Visualization of the membrane surface reconstruction. The arrows show the normals, blue spheres are positions of the marker atoms, and cyan spheres are fitted positions of the markers projected to the approximated quadric surfaces. Voronoi polygons projected onto the quadric surfaces are shown in orange. See the Supporting Information for detailed algorithm description.

Analysis was performed by custom scripts based on the membrane analysis module of Pteros molecular modeling library.^52,53^ The algorithm used in this work is an evolutionary improvement over the previous implementation^42^ with increased accuracy and robustness. A detailed algorithm description is provided in the Supporting Information. An example of the membrane surface reconstruction used for curvature analysis is shown in Figure 2.

#### 2.1.5 Analysis of Ca^2+^ Binding

In order to assess the Ca^2+^ binding to the buckled membranes, all heavy lipid atoms within 0.3 nm of Ca^2+^ ions are identified. The ion is assumed to be in contact with all lipids which are found within this cutoff. The histogram of the count of ion–lipid contacts as a function of mean curvature *K*_m_ of the corresponding lipids *P*_Ca_(*K*_m_) is computed for all trajectory frames. The histogram of mean curvatures of all lipids *P*_lip_(*K*_m_) is also computed.

### 2.2 Bicelle

#### 2.2.1 System Preparation

A pre-equilibrated periodic patch of the POPS bilayer with 128 lipids and 128 K^+^ neutralizing counter ions was employed. The patch was multiplied in the *X*-direction to yield an elongated system with 384 lipids. A slab of water was added at both sides in the *X*-direction to allow for the formation of bicelle caps and in the *Z*-direction to provide enough space for the membrane bending. The final solvation is ∼144 water molecules per lipid. The obtained system was then subjected to an anisotropic pressure coupling along the *Y*-direction only. A short equilibration for ∼10 ns was performed until the edges of the bicelle formed the semi-cylindrical caps.

**Figure 3:**
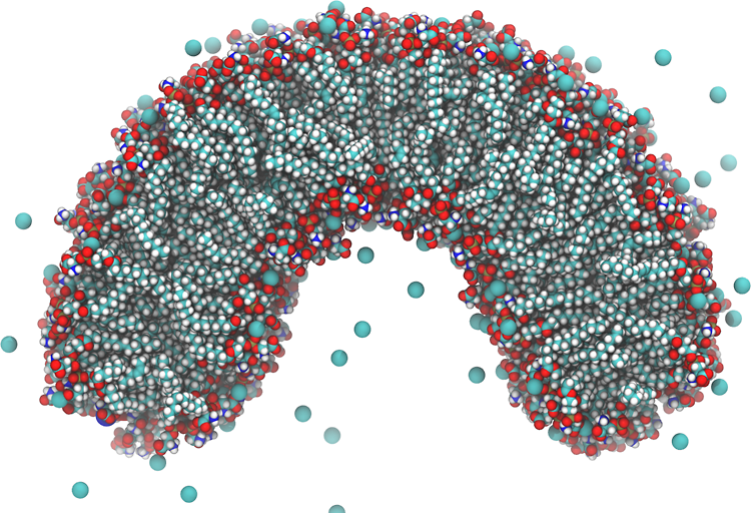
Equilibrated POPS bicelle with enforced curvature of 0.2 nm^–1^. Ca^2+^ ions are shown as spheres, and water is not shown for clarity.

The EnCurv technique^40^ was used to enforce the membrane curvature. The curvature was gradually increased in 0.05 nm^–1^ increments until the target curvature of 0.2 nm^–1^ was reached. The final shape of the curved bicelle is shown in Figure 3.

#### 2.2.2 Production Simulations

The 192 Ca^2+^ ions (1:2 ion-to-lipid ratio) were added by randomly substituting K^+^ counter ions used for initial equilibration. The not substituted K^+^ ions were deleted. No Cl^–^ ions were added. Simulations were performed with prosECCo75 force field^1^ for 300 ns. The last 200 ns of trajectories was used for analysis. Additionally, the same simulations with CHARMM36 with the NBFIX correction for Ca^2+^^28,31^ were performed for force field comparison. Unless indicated otherwise, the results in the main text correspond to prosECCo75 force field.

#### 2.2.3 Analysis of Ca^2+^ Binding

The binding of Ca^2+^ to the concave and convex leaflets of the bicelle was analyzed separately. Only the bicelle’s central bilayer sector is included into the analysis, while the caps and adjacent regions are ignored. Ca^2+^ ion is assumed to be in contact with the lipid if it is within 0.3 nm of any of its heavy atoms. After determining all ion–lipid contacts, they are subdivided into the contacts with three major structural regions: head groups (serine moiety, labeled as “head”), phosphate groups (labeled as “po4”), and carbonyl groups of lipid tails (labeled as “carbo”). If the ion is in contact with several lipids simultaneously, the detailed statistics of the contacting regions of each lipid are recorded in the form of the contact pattern. For example, the pattern “head-head-po4” means that the ion is in contact with the head groups of two lipids and the phosphate of the third lipid. The probabilities of all contact patterns, which occurred during the simulation, were computed. Analysis was performed by the custom scripts based on Pteros molecular modeling library.^9,10^

## 3 Results

### 3.1 Ca^2+^ Binding to the Buckled Membranes

A visual inspection of the trajectories shows that Ca^2+^ ions interact with POPC membranes only transiently without forming strong complexes with the lipids. In contrast, the interaction of these ions with an anionic POPS bilayer is expectantly much stronger, with almost all Ca^2+^ ions bound to the bilayer during the equilibrated part of simulation trajectories.

**Figure 4:**
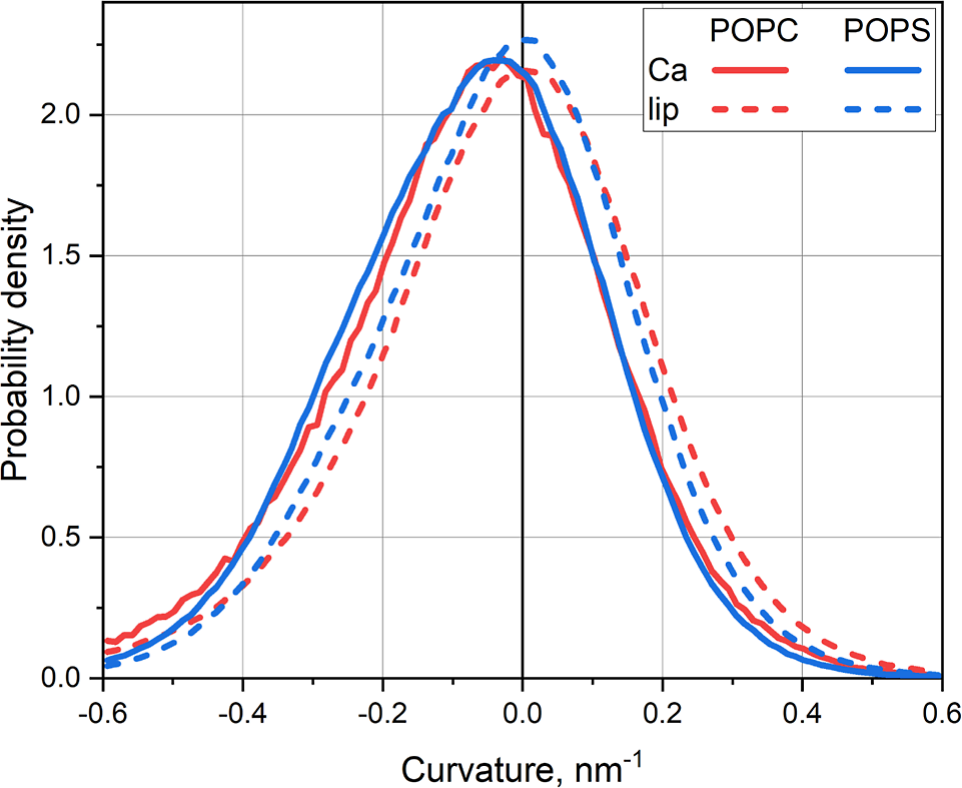
Probability density histograms of finding any lipids, either in contact or not in contact with Ca^2+^ ions (“lip”) or only the lipids in contact with Ca^2+^ ions (“Ca”), in the regions of particular curvature. Results for POPC and POPS bilayers are shown.

The analysis of curvature distributions for all lipids in the system and the lipids interacting with the ions shows that their shape is very similar for POPC and POPS systems (Figure 4).

The histograms for lipids in contact with Ca^2+^ are shifted systematically to the left, which means that Ca^2+^ ions prefer to interact with lipids in the regions of negative curvature (i.e., at concave membrane surfaces). Remarkably, this preference is independent of the absolute interaction strength between Ca^2+^ and the lipids being almost the same for neutral POPC and anionic POPS bilayers.

### 3.2 Ca^2+^ Binding to the POPS Membrane with Enforced Curvature

The details of Ca^2+^ binding to anionic membranes was further studied in simulations with enforced curvature. In this case, we explicitly compared the binding to the concave (negative curvature) and convex (positive curvature) membrane surfaces.

#### 3.2.1 Lipid Coordination by Ca^2+^ Ions

**Figure 5:**
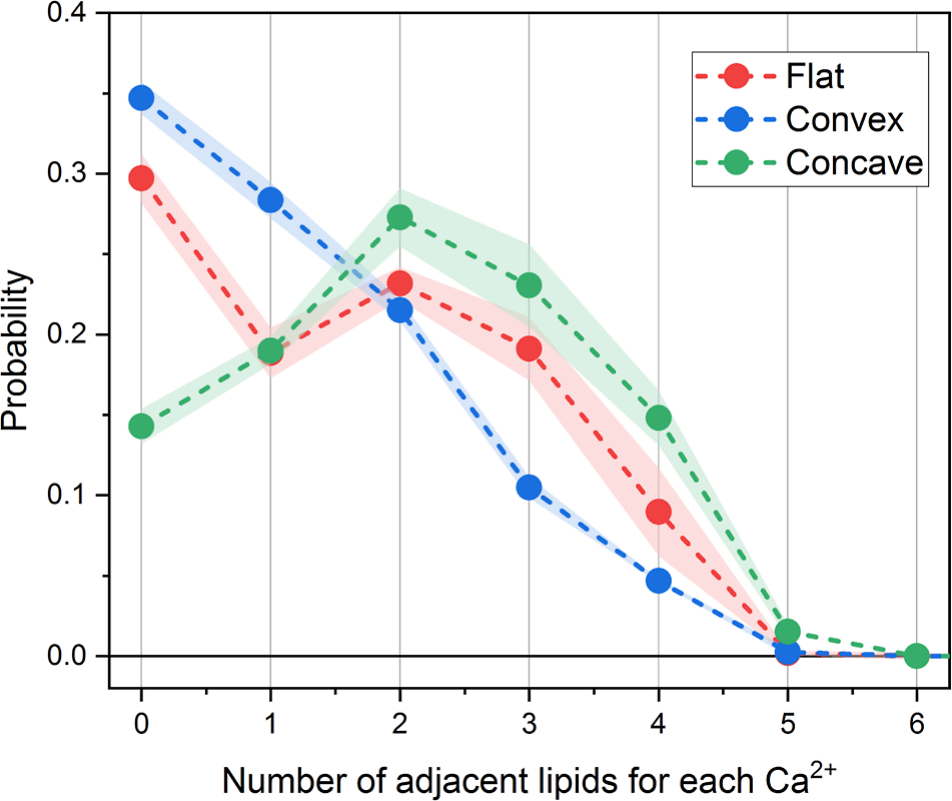
Distributions of the number of lipids, coordinated by the same Ca^2+^ ion for flat, convex, and concave monolayers of POPS membranes. The connecting lines are shown for aiding visual comparison only. The errors are evaluated from the distributions computed independently for three equal non-overlapping parts of the trajectory. The error bars are shown as the semi-transparent bands.

It is well known that divalent Ca^2+^ ions may facilitate coordination and cross-linking of anionic lipids. The magnitude and the curvature dependence of this effect is of interest since it may depend strongly on the distance between the head groups. We computed the distributions of the number of lipids, coordinated by the same Ca^2+^ ion for flat, concave, and convex membrane monolayers (Figure 5).

In the flat membrane, the distribution appears to be bi-modal with peaks at 0 and 2, which means that there is a significant number of free ions and ions that cross-link two POPS lipids. The maximal coordination number observed is 6 (only several frames with such coordination are observed in the trajectory, with a probability of ∼3 × 10^–5^), with the coordination of 1–3 lipids being the most common.

The membrane bending changes this distribution systematically and depending on the sign of curvature. At concave membrane surfaces (negative curvature), the distribution shifts significantly to higher coordination numbers, with the number of free ions dropping to almost half. At convex surfaces, the opposite effect is observed—the distribution shifts to lower coordination numbers, and the number of free ions increases, with the curve decreasing monotonously.

**Figure 6:**
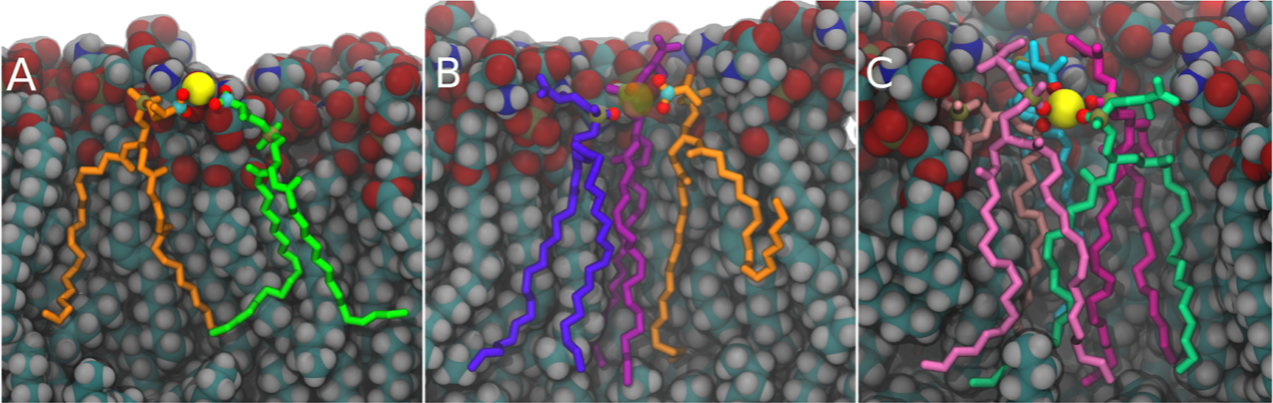
Typical instantaneous structures with Ca^2+^ ions coordinated with two (A), three (B), and five (C) lipids where the latter is rare. The Ca^2+^ ions are shown as yellow space-filled spheres. (B) Ca^2+^ ion is made transparent in order not to obscure the bound lipid behind it. Bound lipids are shown as sticks and colored individually (hydrogens are not shown for clarity). The atoms involved in calcium coordination are shown as small spheres. Other lipids are shown in a space fill representation and colored according to the atom type. All snapshots correspond to the flat membrane. Similar structures observed in the curved membranes are visually indistinguishable.

Typical structures from MD trajectories with Ca^2+^ ions coordinated by multiple lipids are shown in Figure 6. It can be clearly seen that there are multiple modes of binding involving serine head groups, phosphates, or carbonyl moieties of the POPS lipids. Also, the higher the coordination number, the more sterically restrained the involved lipids are. This agrees with the overall rigidification and increases ordering of the membrane, which is known to occur upon calcium binding.

#### 3.2.2 Detailed Binding Patterns

**Figure 7:**
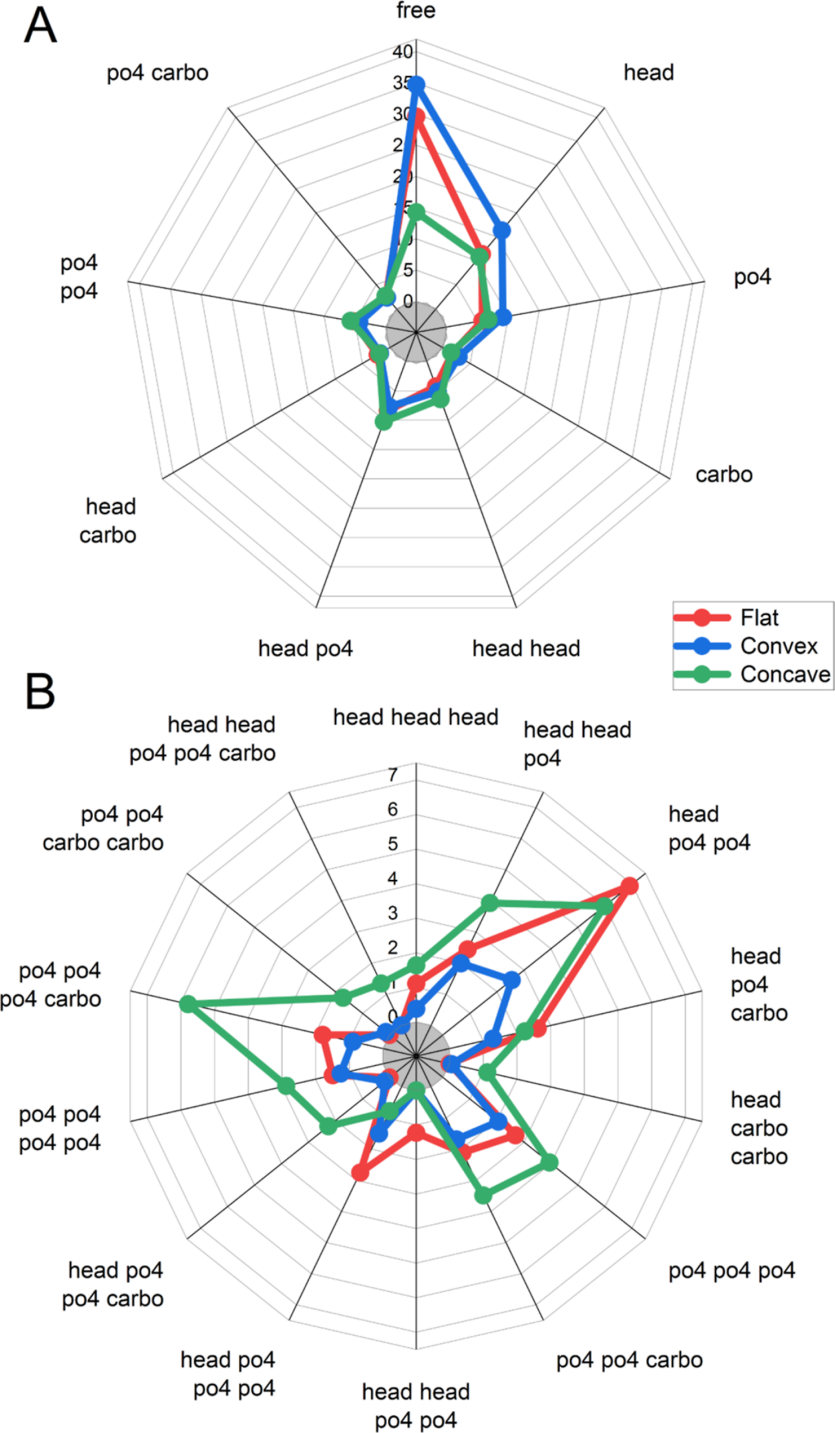
Relative abundance of the binding patterns of Ca^2+^ ions with different chemical groups of coordinated POPS lipids. (A) Low-coordination patterns (between 0 and 2 lipids). (B) High-coordination patterns (3–5 lipids). The scale is in % of abundance among all detected binding patterns in simulations. “Free” stands for uncoordinated free Ca^2+^ ions, “head” for the ions coordinated with serine head group moiety, “phosphate” for the ions coordinated with the PO_4_ phosphate group, “carbo” for the ions coordinated with carbonyl groups of the lipid tails. The abbreviation “po4 po4 carbo” means that the same Ca^2+^ ion is coordinated with two phosphate groups and one carbonyl group, and “head po4 po4 carbo” means that the ion is coordinated with the serine moiety, two phosphate groups, and one carbonyl group. The same logic applies to other patterns.

The coordination of Ca^2+^ ions was studied in more detail by computing the binding patterns of the ions with different coordinating groups of POPS lipids. Figure 7 shows the relative abundances of the binding patterns, which constitute more than 1% of all patterns observed in simulations. Low-coordination patterns (between 0 and 2 lipids) and high-coordination patterns (3–5 lipids) are shown in the separate panels for clarity.

It is evident that the abundance of free Ca^2+^ ions increases near the convex monolayers and decreases near the concave ones (Figure 7A), which is in accordance with the coordination histograms in Figure 6. However, other low-coordination patterns appear to be almost insensitive to the curvature (Figure 7A). Only “head” and “po4” patterns become slightly more abundant in the convex monolayers. In contrast, binding patterns with two lipids do not change at all, regardless of the curvature. This suggests that cross-linking of two lipids by the calcium ions is a very robust phenomenon relatively insensitive to the curvature-related changes of the lipid packing.

Nevertheless, high coordination patterns appear curvature-dependent despite being less abundant. The general trend for them is the opposite to one observed for free calcium; their abundance increases in concave monolayers and decreases in the convex ones.

For the flat membrane, the dominant pattern is “head po4 po4”, while the patterns involving the carbonyls are rarely observed. In the convex monolayers, the abundances of all patterns involving head groups decrease dramatically, while the patterns which involve only phosphates and carbonyls are much less affected. In the concave monolayers, a more complex picture is observed. The pattern “head po4 po4” is similarly pronounced for the flat membrane, while the abundance of all patterns involving only phosphates and carbonyls increases. This effect is especially pronounced for the pattern “po4 po4 po4 carbo”, which becomes the most abundant in concave monolayers. There is also an increase of patterns with five or six coordinated lipids (such as “head head po4 po4 carbo”).

### 3.3 Influence of Charge Scaling on Ca^2+^ Binding

**Figure 8:**
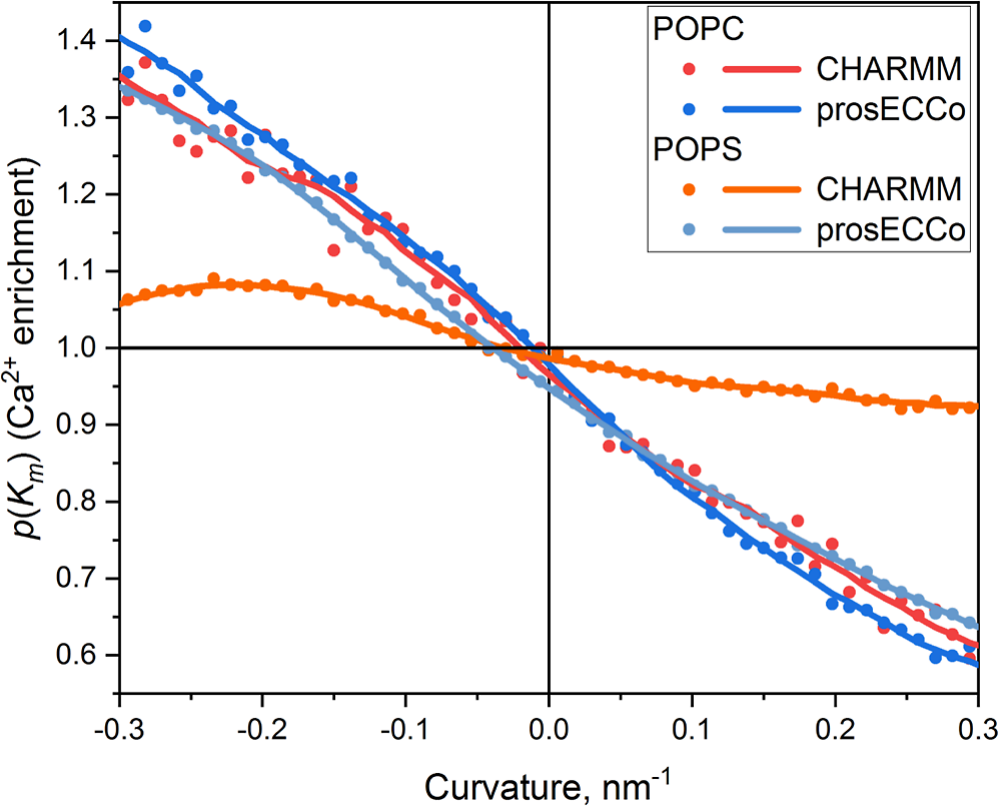
Enrichment plots for POPC and POPS bilayers. The values *p*(*K*_m_) *>* 1 mean that Ca^2+^ ions prefer to bind to these regions of the membrane, *p* < 1 means that the Ca^2+^ ions avoid such regions, and *p*(*K*_m_) = 1 means that there is no preference in binding in comparison to the flat membrane.

In order to elucidate the influence of charge scaling on Ca^2+^ binding, we compared full charge CHARMM force field with the scaled charge prosECCo75 force field applied to the same buckled POPC and POPS systems. We computed the ion enrichment plots by dividing the curvature distributions for Ca^2+^-bound lipids over the distributions of all lipids: . Individual distributions, which were used for this analysis, are shown in Figure S1. If *p*(*K*_m_) > 1, then Ca^2+^ ions prefer to bind to the regions with the given curvature, if *p*(*K*_m_) < 1, the Ca^2+^ avoid such regions, and if *p*(*K*_m_) = 1, there is no preference in binding. Enrichment plots are shown in Figure 8.

It can be clearly seen that there is a preference of Ca^2+^ binding to negatively curved (concave) parts of the membrane surface in all studied systems; however, the magnitude of this effect depends on the force field. The enrichment curves for POPC with both CHARMM and prosECCo75 force fields and the curve for POPS with prosECCo75 are almost identical. In contrast, the magnitude of enrichment for POPS with CHARMM force field is much smaller.

In the case of prosECCo75 force field, the curvature preference is identical for neutral POPC and anionic POPS bilayers despite a dramatic difference in the binding energy itself (Figure S2). The CHARMM force field shows similar curvature preference for neutral POPC bilayer as in prosECCo75. However, in the case of anionic POPS bilayer, compared to prosECCo75 results, CHARMM force field overestimates the binding energy to the extent, which makes the influence of curvature almost negligible. We have also compared for CHARMM and prosECCo75 force fields average energies of Ca^2+^ binding to different chemical groups of POPS lipids (Figure S2), histograms of lipid coordination number (Figure S3), and the binding patterns (Figure S4). The results of this comparison confirm that CHARMM force field not only significantly increases the overall Ca^2+^ binding to anionic lipids but also changes the number of coordinated lipids and the coordination patterns with different chemical groups. All this makes for CHARMM Ca^2+^ binding much less sensitive to the influence of the membrane curvature for POPS. A more detailed discussion of these results is presented in the Supporting Information.

We also analyzed the distributions of the residence times of Ca^2+^ ions, which are coordinated by the POPS lipids. The convex and concave monolayers were considered separately, and CHARMM and prosECCo75 force fields were compared. The results are shown in Figure S5. There are no detectable differences in the residence time distributions caused by either the monolayer curvature or the particular force field used.

## 4 Discussion and Conclusions

Curved membranes are of great interest in terms of their binding to ions in general and Ca^2+^ in particular. In addition to straightforward biological implications related to calcium signaling and its influence on the membrane proteins, curved membranes provide a unique geometrically heterogeneous environment for studying calcium binding. Indeed, curvature changes the distance between the lipid groups involved in the ion binding, such as head groups, phosphates, and carbonyls. In concave monolayers, lipids are congested, creating an environment favorable for higher coordination or ions. In contrast, in convex monolayers, lipid heads are further apart, thus favoring smaller coordination numbers and binding to individual lipids.

The binding of Ca^2+^ ions to the curved lipid bilayers remains poorly studied despite significant progress in realistic membrane simulations. There are two factors, which hampered such studies up to now. First, until recently, there were no tools available for simulating lipid membranes with enforced uniform curvature. Existing simulation techniques produced systems with variable curvatures, which complicated the analysis and made it hard to isolate the effect of curvature from elastic deformations, mechanical stress, and surface tension fluctuations. Introducing techniques for enforcing membrane curvature in strain-free lipid bilayers^40^ allowed for overcoming these problems. Second, the widely used non-polarizable force fields tend to overestimate Ca^2+^ binding to anionic moieties, making them hardly usable for quantitative simulations of spatially confined and polycoordinated systems such as anionic lipid bilayers. Using charge scaling mitigates the problem of calcium overbinding without the loss of computational efficiency.

In this work, we combined recently developed techniques for enforcing uniform membrane curvature with a charge scaled force field to systematically study of Ca^2+^ binding to curved lipid bilayers containing either neutral POPC or anionic POPS lipids. We first studied the binding of Ca^2+^ ions to the buckled elastically bent membrane, which fluctuates freely within the constraints imposed by the simulation box and exhibits the range of transient curvatures changing in time and space. We observed that Ca^2+^ ions interact preferentially with the concave regions for both neutral POPC and anionic POPS membranes. The relative magnitude of this preference increases with the increase of mean membrane curvature and is remarkably identical for POPC and POPS membranes, despite very different binding energies of Ca^2+^ ions.

Next, we studied the binding of Ca^2+^ to a POPS membrane with enforced curvature. This allowed us to gather enough statistics for analyzing possible patterns of Ca^2+^ binding with different chemical groups of multiple coordinated lipids. The following findings were made:

1. The amount of free calcium increases near the convex membrane surfaces and decreases near the concave ones.
2. Although Ca^2+^ ions most often cross-link two POPS lipids, a significant number of complexes reaches up to five-coordinated lipids.
3. The relative abundance of Ca^2+^ ions coordinated with one or two lipids is not sensitive to curvature. In contrast, the abundance of highly coordinated Ca^2+^ ions (bonded to three or more lipids) decreases in the convex monolayers and increases in the concave ones.
4. Multiple coordination of Ca^2+^ ions to serine head group moieties decreases dramatically in the convex monolayers but does not change substantially in the concave ones. In contrast, multiple coordination involving only phosphate and carbonyl moieties does not change significantly in the convex monolayers but increases considerably in the concave ones.

This behavior can be explained in terms of changes of distances between the lipids in the convex vs concave monolayers. In concave monolayers, lipids are more congested which favors polycoordination involving phosphate and carbonyl groups of adjacent lipids which are close enough to interact with the same Ca^2+^ ion. The head groups are not affected much since their contacts with the ions are already mostly saturated and bringing them even closer together has little effect. In convex monolayers, the distance between the lipids increases the most at the head group region since it is the farthest from the center of curvature. That is why polycoordination of head groups decreases significantly while contacts with phosphate and carbonyl groups, which are located deeper in the membrane, are barely affected.

Finally, we compared results of simulations performed with the full charge CHARMM force field to those of the scaled charge prosECCo75 force field. We have found multiple differences caused by much stronger Ca^2+^ binding to anionic POPS lipids within former approach. Particularly, the curvature-related binding preferences almost disappear for POPS lipids when the CHARMM force field is used (Figure 8). We attribute this to Ca^2+^ overbinding, which overrides the rather subtle curvature effects. Also, the lipid coordination distributions and the curvature-dependent changes of the binding patterns are affected by the use of the full charge force field (see the Supporting Information for details). This interpretation is confirmed by the fact that there is no detectable differences between CHARMM and prosECCo75 force fields for the neutral POPC lipids (Figure 8). In this case, the absence of negative charges eliminates the problem of excessive electrostatic attraction between Ca^2+^ and the lipid atoms; consequently, the two force fields produce indistinguishable results. In the case of negatively charged POPS lipids, the absence of polarization screening effects in the non-polarizable force field leads to severely exaggerated Coulombic attraction between the charges. In contrast, scaling in prosECCo75 force field reproduces the screening effects, moreover, in a way that does not require significant force field modifications or unphysical assumptions.

## Supporting Information Available

Algorithm of curvature analysis; detailed comparison of Ca^2+^ binding in CHARMM and prosECCo75 force fields (curvature histograms for buckled membranes, binding energies for enforced curvature membranes, histograms of lipid coordination by Ca^2+^ ions, and detailed binding patterns); and analysis of Ca^2+^ residence time (PDF)
