# Supplementary material for: Curvature Matters: Modeling Calcium Binding to Neutral and Anionic Phospholipid Bilayers
Source: J Phys Chem B. 2023 May 16;127(20):4523–31. doi: 10.1021/acs.jpcb.3c01962 (PMC10226117; doi:10.1021/acs.jpcb.3c01962)
Supplement: Supplementary file 1 — jp3c01962_si_001.pdf [file jp3c01962_si_001.pdf]

Curvature matters: Modelling Calcium Binding to Neutral and Anionic Phospholipid Bilayers

Supplementary Information

Semen Yesylevskyy [1,2,3,*], Hector Martinez-Seara [1], Pavel Jungwirth [1]

[1] Institute of Organic Chemistry and Biochemistry, Academy of Sciences of the Czech Republic, Flemingovo náměstí 542/2, 160 00 Prague 6, Czech Republic;
[2] Department of Physics of Biological Systems, Institute of Physics of The National Academy of Sciences of Ukraine, Nauky Ave. 46, 03038, Kyiv, Ukraine;
[3] Receptor.AI Inc., 20-22 Wenlock Road, London N1 7GU, United Kingdom.

[*] Corresponding author: yesint4@gmail.com

The algorithm of curvature analysis

Each lipid is represented by the marker point, which is set to the position of phosphate atom. The following steps are perfromed on each MD trajectory frame:

1. The lipid markers within the local patch of radius *r* are selected around each lipid (*r* = 1.7 nm in this study).
2. The inertia axes of the patch are computed and used as a local coordinate system with local Z coordinate (the direction of smallest moment of inertia) being the estimated membrane normal.
3. The direction of the normal is first corrected according to the tail-to-head vector of the central lipid of the patch (the normal is assumed to point outwards from the bilayer center). If this normal is still more than 45° inclined to the normals of any two lipids in the patch within 1.0 nm from the central lipid, then it is reset to the average of normals of these adjacent lipids. This error correction procedure allows to avoid incorrect normal orientations in the regions of extreme instantaneous curvature, where the inertia axes of the patch could give an unreliable estimate of the normal direction.
4. Positions of all markers in the patch are computed in the local coordinates x_l, y_l, z_l and the quadric surface $z_l = Ax_l^2 + By_l^2 + Cx_l y_l + Dx_l + Ey_l + F$ is fitted to them using the mean-squares fitting.

5. True normals are computed as the normal vectors to the fitted quadric surface at the position of the central marker. Mean and Gaussian curvatures are computed analytically from the I and II fundamental forms (see (1) for details).
6. Markers are projected into the true tangent plane and 2D Voronoi diagram is computed for the marker of the central lipid. Nearest neighbor lipids are detected as direct Voronoi neighbors.
7. Vertices of the Voronoi polygon are projected back into the fitted quadric surface and the area per lipid is computed by summing up the triangular surface faces around the central atom.
8. Triangulated membrane surface is computed is requested (for the visualization purpose only).

Comparison of Ca^{2+} binding in CHARMM and prosECCo75 force fields

Curvature histograms for buckled membranes

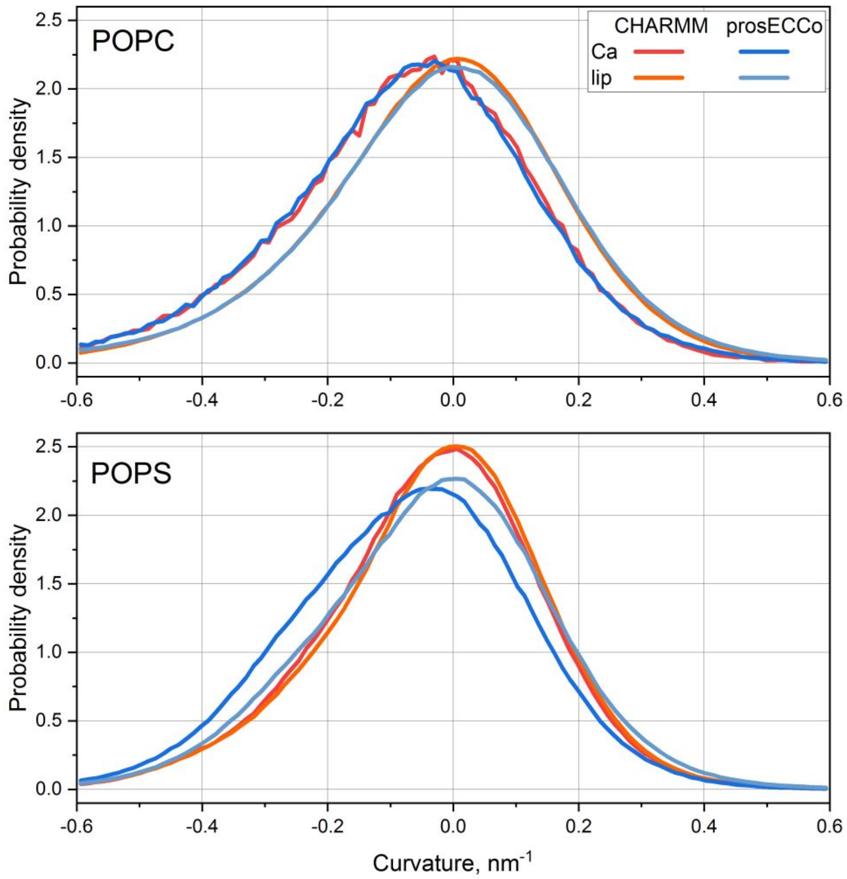

Figure S1. Probability density histograms of finding any lipids (lip) or the lipids in contact with Ca^{2+} ions (Ca), in the regions of particular curvature. The results for POPC (top panel) and POPS (bottom panel) bilayers are shown for CHARMM and prosECCo75 force fields.

In prosECCo75 the histograms for lipids in contact with Ca^{2+} are shifted systematically to the left, which means that Ca^{2+} prefer to interact with lipids in the regions of negative curvature (concave membrane surface). There is no such shift for POPS if the CHARMM force field is used (Fig. S1). This is a clear indication of severe Ca^{2+} overbinding to anionic lipids, which overrides the effect of curvature.

Binding energies for enforced curvature membranes

The average binding energies of Ca^{2+} ions with different chemical groups of POPS lipids were computed (Fig. S2). The binding in CHARMM is systematically stronger than in prosECCo75. This difference depends on the net charge of the chemical group the ions are interacting with. It is the largest for serine head groups followed by phosphates and carbonyls. There is no difference between the monolayers of flat membranes (they are shown as "convex/concave" for the flat membrane in figure S2 only for convenience of comparison), but the binding to concave monolayers of the curved membranes is systematically stronger for both force fields and all three chemical groups. The absolute magnitude of this curvature-dependent effect is comparable in CHARMM and prosECCo75 force fields, but considering weaker overall binding in prosECCo75 the relative influence of curvature is stronger in prosECCo75.

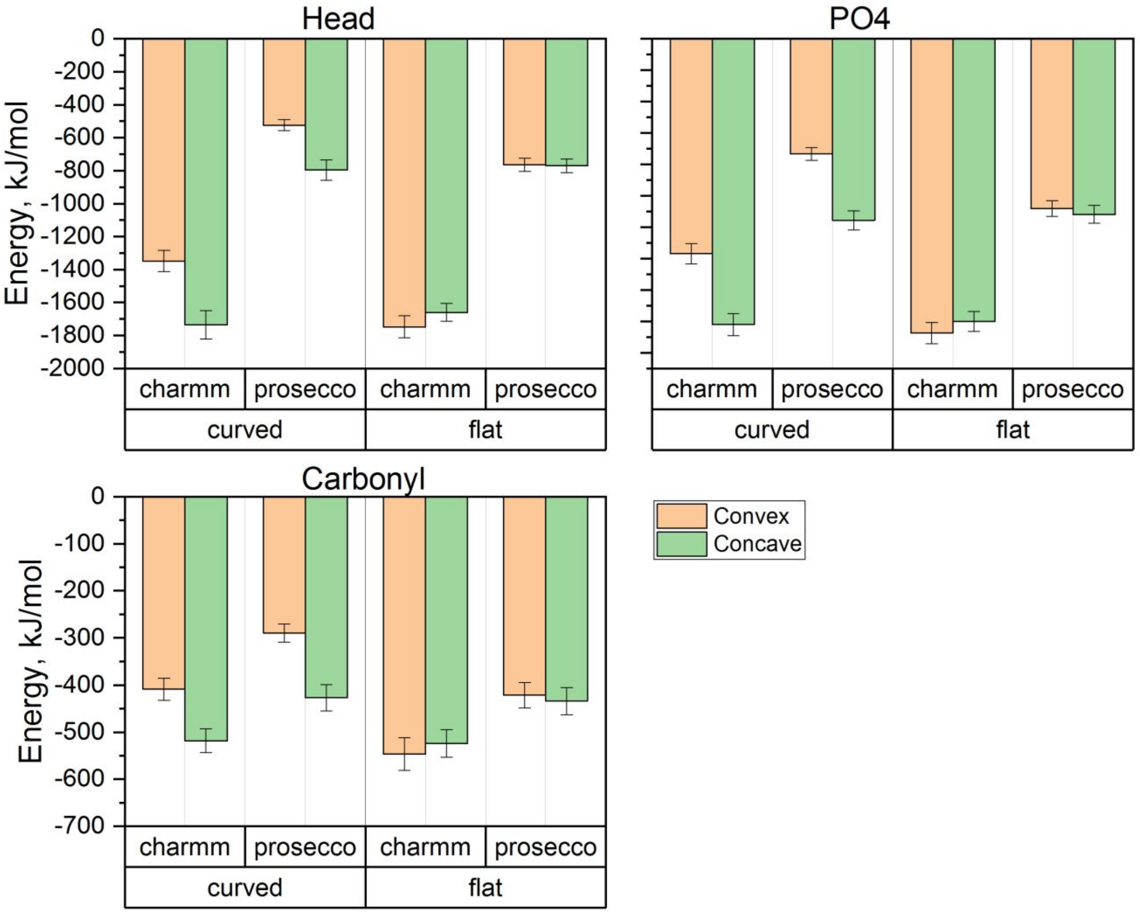

Figure S2. Average binding energies between Ca^{2+} ions and different chemical groups of POPS lipids. For the flat membrane the monolayers marked as "convex" and "concave" correspond to actual convex and concave monolayers in the curved system.

Lipid coordination by Ca^{2+} ions

We computed histograms of the number of lipids, coordinated by the same Ca^{2+} ion in CHARMM and prosECCo75 force fields (Fig. S3).

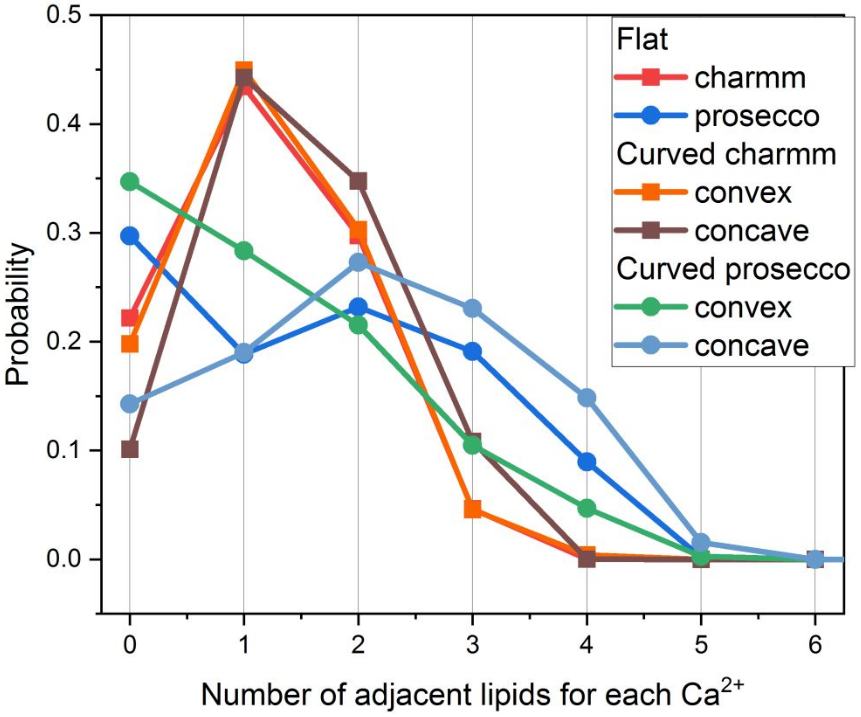

Figure S3. Histograms of the number of lipids, coordinated by the same Ca^{2+} ion.

In the CHARMM force field the dominant number of lipids coordinated by the same ion in the flat membrane is 1. The distribution is rather narrow and the coordination numbers higher than 3 are not observed at all. In contrast, in prosECCo75 the distribution is much broader with the peaks at 2 and 0, which means that there is a significant number of free ions and the ions, which cross-link two POPS lipids. The maximal coordination number observed is 6 and the coordination of 3-5 lipids is rather abundant.

The impact of curvature is also different in different force fields. In CHARMM the effect of curvature is minor. There is no difference in coordination between flat and convex membrane surfaces while the distribution for concave surface is shifted slightly to the right and the number of free ions decreases. In contrast, in prosECCo75 the influence of curvature is much more pronounced. In the concave membrane surfaces the distribution shift to the right significantly and the number of free ions drops. In the convex surface the opposite effect is observed – the distribution shifts to the left and the number of free ions increases.

Detailed binding patterns

The binding patterns of Ca^{2+} ions with different chemical groups of POPS lipids computed in CHARMM and prosECCo75 force fields are shown in Fig. S4.

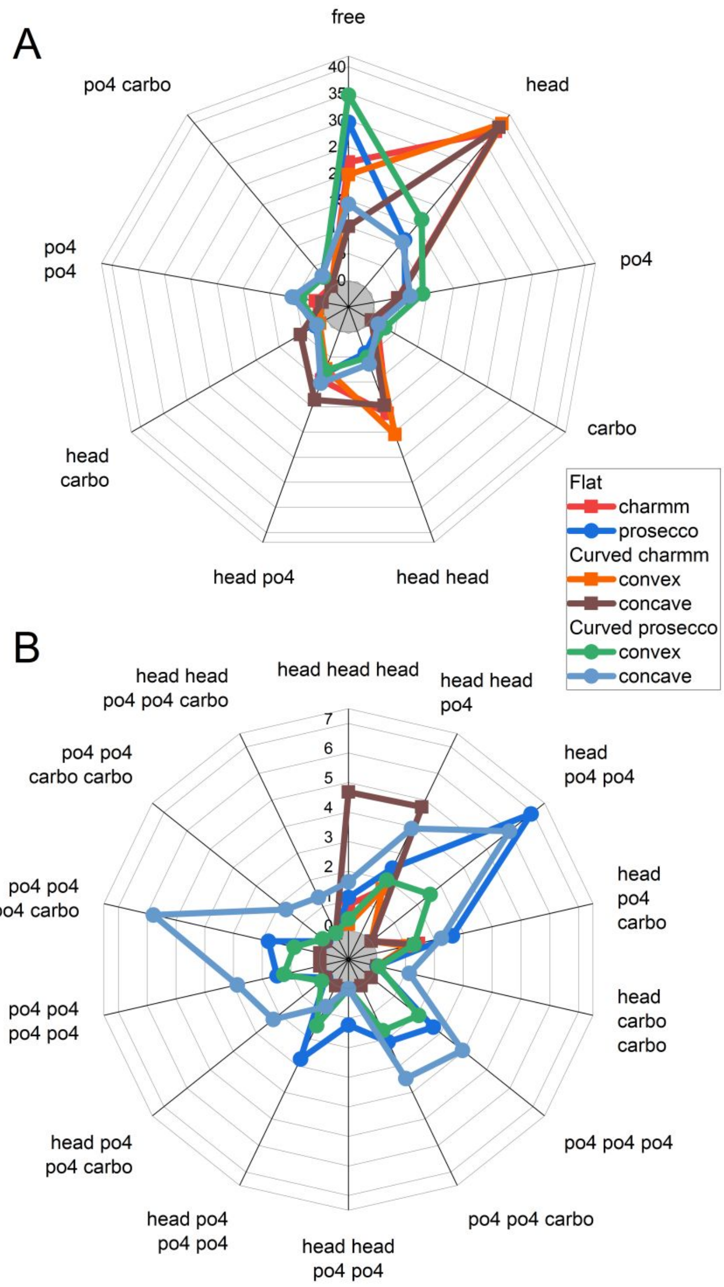

Figure S4. The relative abundance of the binding patterns of Ca^{2+} ions with different chemical groups of coordinated POPS lipids. Panel A shows low-coordination patterns (between 0 and 2 lipids). Panel B shows high-coordination patterns (3-4 lipids). “Free” stands for uncoordinated free Ca^{2+} ions. The scale is in % of abundance among all detected binding patterns in simulations.

In the case of CHARMM force field the dominant pattern is binding to a single head group (“head” in Fig. 8A) with some contribution of two head groups (“head head” in Fig. S4A). The influence on curvature is only visible for concave monolayers. In this case the abundance of free ions decreases and the patterns “head po4”, “head carbo” appear. Two high-coordination patterns (“head head po4” and “head head head” in Fig. S4B) also become more abundant in concave monolayers. It is remarkable that for CHARMM serine head groups are always involved in binding, while the phosphate are only rarely involved and the binding to carbonyls is almost never observed.

In the case of prosECCo75 force field the picture is dramatically different. The abundance of low coordination patterns is much lower (except the free ions) in comparison to CHARMM. The curvature induces the opposite changes in the abundance of free ions. 7. The high coordination patterns are much more abundant in comparison with CHARMM. In the flat membrane the binding involving at least two phosphates dominates ("head po4 po4", "po4 po4 po4", "head po4 po4 po4" in Fig. 8B). In the convex monolayer "head po4 po4" patterns decreases a lot, while the rest decrease slightly or does not change. In the concave monolayer a dramatic increase of the pattern "po5 po4 po4 carbo" is observed followed by a moderate increase of other patterns involving phosphates and carbonyls of 4 and 5 lipids (Fig. 8B). In prosECCo75 the binding always involves phosphate and carbonyl groups, while the binding to head groups is rare, which is in striking contrast with CHARMM.

Ca^{2+} residence time analysis

The residence time was computed in terms of residue-level contacts. Each ion-lipid pair was considered as a distinct entity in the analysis regardless of possible multiple coordination. A contact was considered formed using the same criteria as for the binding patterns analysis above. The convex and concave monolayers were considered separately and the CHARMM and prosECCo75 force fields were compared. The results are shown in Fig. S5.

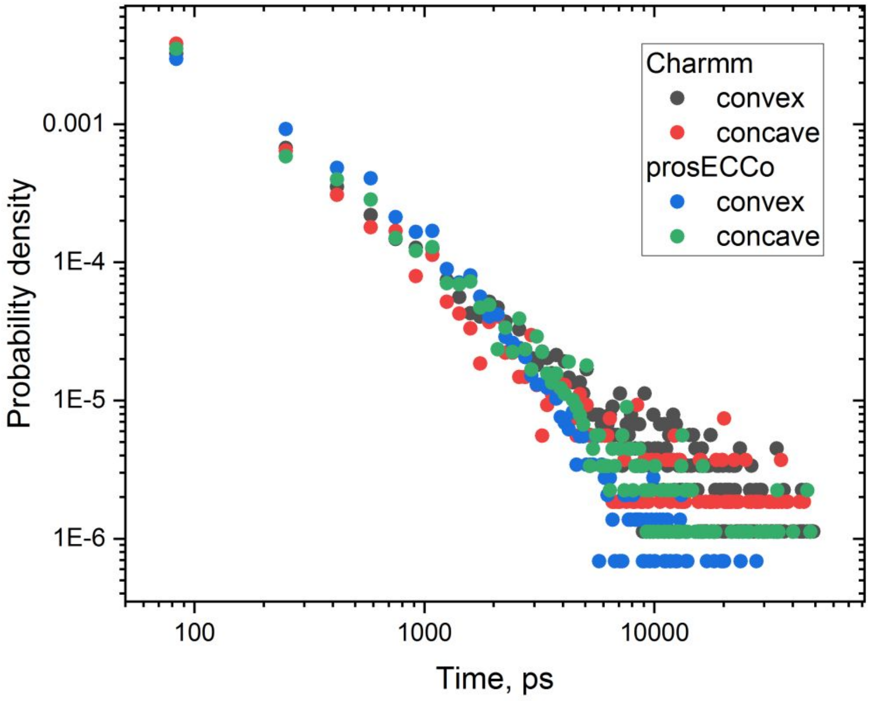

Figure S5. The residence time distributions of Ca2+ ions in contact with POPS lipids.

The residence times show a complex non-exponential distribution with no detectable differences caused by either the monolayer curvature or the force field used. All curves overlap within the limits of data points scatter.

References

1. Yesylevskyy SO, Ramseyer C. Determination of mean and Gaussian curvatures of highly curved asymmetric lipid bilayers: the case study of the influence of cholesterol on the membrane shape. Phys Chem Chem Phys PCCP. 2014 Aug 28;16(32):17052–61.
